# ‘Before and After’. The Journey of Patients With Low Back Pain Consulting in Elective Spine Surgery Clinics. A Qualitative Study Protocol

**DOI:** 10.1111/hex.70301

**Published:** 2025-05-20

**Authors:** Matthew Fernandez, Deb Lees, Katie de Luca, Dawn Dane, Peter Stilwell, Leanda McKenna, Brigitte Tampin, Caroline Bulsara, Tamar Pincus, Michelle Kendell

**Affiliations:** ^1^ Discipline of Chiropractic CQUniversity Brisbane Australia; ^2^ School of Physical and Occupational Therapy McGill University Montreal Canada; ^3^ Department of Sports Science and Clinical Biomechanics, Research Unit of Movement, Culture and Society University of Southern Denmark Odense Denmark; ^4^ South Metropolitan Health Service, Fiona Stanley Fremantle Hospital Group, Department of Physiotherapy Perth Australia; ^5^ Curtin School of Allied Health, Faculty of Health Sciences Curtin University Perth Australia; ^6^ North Metropolitan Health Service Sir Charles Gairdner Hospital, Department of Physiotherapy, Neurosurgery Spinal Clinic Perth Australia; ^7^ Hochschule Osnabrueck, Faculty of Business and Social Sciences Osnabrueck University of Applied Sciences Osnabrueck Germany; ^8^ Institute for Health Research & National School of Nursing & Midwifery The University of Notre Dame Notre Dame Indiana USA; ^9^ Department of Psychology University of Southampton Southampton UK

**Keywords:** care journey, low back pain, patient expectations, patient experience, qualitative research, surgery

## Abstract

**Introduction:**

Low back pain (LBP) is a highly prevalent and disabling condition. People with LBP may be referred to elective surgical clinics for further evaluation and consideration of surgery. Despite long waits for an initial appointment, many of these patients are not surgical candidates and may be discharged, receiving minimal‐to‐no care, advice or alternative treatment options, leaving a critical gap in care.

**Methods:**

Approximately 25 participants with dominant, chronic non‐specific axial LBP who are (1) referred to and (2) discharged without spinal surgery following consultation in two elective spinal surgery clinics in Western Australia will participate in a one‐on‐one pre‐consultation semi‐structured interview and a similar post‐consultation qualitative interview. Purposive sampling will be used to recruit participants. Interviews will be audio‐recorded and transcribed, underpinned by a qualitative descriptive approach to explore participants' care journey, including pre‐consultation expectations and overall experiences post‐consultation. We will use inductive content analysis to analyse our data, allowing for the identification of themes that are generated from the participants' responses.

**Discussion/Conclusion:**

This protocol outlines the methodological process for a qualitative study exploring the experiences and expectations of LBP patients before and after consulting in elective spinal surgery clinics in Western Australia. The findings may give rise to consumer‐focused solutions to improve the care journey and highlight gaps in patient expectations and understanding of non‐surgical management, informing the development of tailored educational resources, communication strategies and new care pathways.

**Patient or Public Contribution:**

This study will incorporate patient and/or public involvement by engaging representatives from Musculoskeletal Australia (https://muscha.org/) to contribute to the study design and interpretation of findings. Specifically, a consumer with lived experience of low back pain will be invited to review and provide feedback on the semi‐structured interview questions, to ensure they are appropriate, accessible and reflective of patient experiences.

**Clinical Trial Registration:**

This study is not a clinical trial and is therefore not registered.

## 1 Introduction

Low back pain (LBP) is an extremely common condition and is among the leading causes of disability in Australia (‘Back problems. Australian Institute of Health and Welfare’, [1]), with an estimated 2.7 million people reporting the condition nationally in 2019 [2]. LBP often causes limitations to many day‐to‐day activities, reducing one's quality of life [3], with more than 50% of people with LBP seeking care annually [4]. Clinical practice guidelines endorse interventions, such as advice to stay active, reassurance and education about LBP, and recommend psychological and physical treatment, like exercise, as part of the self‐management care for LBP [5, 6]. Guidelines further recommend judicious use of pain medication and less emphasis on surgical interventions [6, 7]. For various reasons, including a lack of strategies to guide clinicians in relevant care options for patients [8], there is a suboptimal and delayed uptake of evidence‐based practice. This leads to the increased use of invasive healthcare resources and associated rising costs with unnecessary, and potentially harmful, treatments that may not be required, such as surgery [5].

For LBP with associated radicular leg pain, decompression (i.e., removing a portion of bone and/or herniated disc material) and fusion (i.e., joining two or more vertebrae together, rendering it immobile) are the most common surgeries performed [9]. These surgical procedures may be indicated in select patients when conservative care fails. Despite only a small percentage of patients, that is, less than 30%, attending elective spinal surgery clinics being considered as appropriate surgical candidates [10, 11, 12], the number of surgical procedures on the spine is increasing. This is despite the lack of sufficiently strong evidence supporting the benefits of spinal fusion surgery over non‐surgical treatments [13]. For instance, between 1997 and 2006, there was a 167% increase in spinal fusion surgery within the private sector in Australia [14]. More recent data showed that, in comparison to the public sector, there has been a disproportionate increase in the number of privately funded elective spinal fusions in Australia between 2001 and 2020 [15]. Apart from the sharp increase in surgical rates, LBP patients are inappropriately referred to surgical settings for further investigation and the possibility of surgery, thereby contributing to lengthy waiting lists for consultation for those more likely to benefit from surgery [16].

Little is known in the literature regarding the care journey of patients, including their expectations and experiences surrounding consultation in elective spinal surgery clinics. Existing evidence identifies several drawbacks in spinal surgery care, including a desire for reduced waiting times by patients and a need for ongoing care and support with appropriate information shared in a timely manner [17]. Other drawbacks also include being dismissed or pain being overlooked [18], as well as a lack of reassurance and how to self‐manage ongoing LBP and disability [19]. Additionally, patients seek validation of their pain, which is critical to processing and responding to new information [20]. A lack of validation may see patients reconsult, continuing the cycle of care and cure seeking [21] and maintaining high levels of healthcare utilisation [20], having felt dissatisfied with the explanation and proposed care or advice that they received in the consultation [19]. Importantly, those consultations might be the last contact for patients with healthcare providers within a specialty spinal pain service. As a result, patient needs may not be adequately met and may correlate with unhelpful beliefs about LBP, as well as potentially misguided care expectations within the health system [22].

To our knowledge, no data in Australia exists with respect to the care journey and experiences of LBP patients referred to and consulting in elective spinal surgery clinics. Patient expectations pre‐consultation, including an understanding of their LBP, and how their perspectives change post‐consultation are also unknown. Given that a large proportion of LBP patients who are referred to elective spinal surgery clinics are ultimately discharged without a surgical solution, an important gap in care delivery remains. In response, we aim to explore the expectations and experiences of patients with dominant, chronic non‐specific axial LBP, both pre‐ and post‐consultation, who are (1) referred to and (2) discharged without spinal surgery in two elective spinal surgery clinics in Perth, Western Australia. Findings could provide key insights to improving the patient experience and care journey for those not undergoing spinal surgery.

## 2 Materials and Methods

### 2.1 Study Design and Framework

This study is qualitative in design using semi‐structured one‐on‐one interviews, pre‐ and post‐consultation, and is underpinned by a *qualitative descriptive* approach [23], to explore in‐depth information about the individual participant's knowledge, expectations and overall experiences. Qualitative description is often used when there is a dearth of knowledge within a subject area, and detailed descriptions are sought. The focus is on the rich descriptions of participants directly regarding their perspectives.

Qualitative description differs from other qualitative approaches in that it does not aim for thick description (ethnography), theory development (grounded theory) or an interpretive meaning of experience (phenomenology) [24]. As such, this approach is not bound by pre‐existing philosophical or theoretical commitments [25], making it appropriate for this exploratory study, where little is known qualitatively about the topic. Although qualitative description has low levels of interpretation and aims to summarise participants' descriptions, we will log our reflections, assumptions and decision‐making throughout the research process for transparency; priority will lie in participants' descriptions of the phenomena over the researcher's analysis.

### 2.2 Study Setting and Recruitment

Participants will be recruited from the Neurosurgery Spinal Assessment Clinics at Fiona Stanley Hospital (FSH) and Sir Charles Gairdner Hospital (SCGH) in Perth, Western Australia. These elective spinal surgery clinics are led by Advanced Scope Physiotherapists (ASPs) who examine patients referred with spinal pathology for their suitability for spinal surgery, instigate work‐up where required and refer onward for surgical evaluation or appropriate non‐surgical management. Purposive sampling (based on specific characteristics/criteria) will be used to recruit a heterogeneous sample of participants pre‐consultation. This non‐probability sampling technique is appropriate for a qualitative study rather than random sampling, as researchers predefine which types of participants or cases (e.g., participants with LBP referred to and later discharged without surgery) hold the greatest amount of personal experience of the research topic.

### 2.3 Pre‐Consultation Participant Selection and Sample Size

To facilitate participant recruitment, the site Principal Investigator (PI) and/or neurosurgery clerk at FSH and the neurosurgery clerk at SCGH will post a study flyer to potential participants as soon as they have been booked in and informed of their initial consultation appointment. Patients are usually informed of their upcoming initial consultation by letter 30 days before their appointment date. All patients booked for initial consultation will receive the study flyer during the recruitment period to minimise selection bias. The potential participant will initiate contact with the Chief Principal Investigator (CPI) by email, telephone or short message service (SMS) to express their interest in participating in the study. Subsequently, the CPI will arrange a time to conduct an initial telephone screen to confirm eligibility against a ‘standardised eligibility screening checklist’. A Participant Information and Consent Form (PICF), along with a link to an electronic consent form and demographics questionnaire, will be provided by email or a paper copy sent by post to those eligible for the study, and informed consent will be sought. Recruitment is expected to take 5 months to reach the target sample size of 25 pre‐consultation interviews. The final recruitment number, however, will be determined by factors including the level of depth and detail each interview offers in relation to our aim, that is, more or fewer participants may be needed to gather relevant information than the proposed 25 [26]. Based on current discharges at FSH and SCGH, we anticipate that approximately half of the participants who complete the pre‐consultation interview will meet the eligibility criteria for the post‐consultation interview. Once recruitment for the pre‐consultation interview is completed, the post‐consultation interview sample size will be constrained to what is possible from the pre‐consultation interview sample size.

The target sample size for the pre‐consultation interview appears feasible. The ASP‐led Neurosurgery Spinal Assessment Clinics book (on average) 300 new patients per month between the two sites. More than half of these are for lumbar spine complaints. Therefore, 65% may be considered as LBP patients (*n* = 195 per month). Conservatively, if 5% (*n* = 10) of these patients contact the CPI expressing interest in participating and approximately half (*n* = 5) are eligible and consent to participate, a sample size of 25 (or more) for the pre‐consultation interview is feasible over the recruitment period.

### 2.4 Eligibility Screening

Screening for study eligibility will be over the telephone using a standardised eligibility screening checklist at a time agreed upon with the potential participant. Screening will be conducted by the CPI, who is a registered healthcare professional and who will ask the potential participant a series of questions to determine if they meet the pre‐consultation eligibility criteria. If deemed eligible, further information about the study will be provided verbally over the telephone. If still interested in participating, the CPI will confirm contact details (email address if available and if not, preferred postal address plus preferred contact phone number) to facilitate the consenting and participation process.

### 2.5 Eligibility Criteria Pre‐Consultation Interview

The pre‐consultation eligibility criteria aim to identify people via telephone screening who are most likely to be discharged without surgery (i.e., those with non‐dominant spine‐related leg pain with or without ‘soft’ neurological symptoms).

#### 2.5.1 Inclusion Criteria

a. Adults (aged ≥ 18).
b. LBP of at least 3‐month duration.
c. Primary presenting complaint is dominant, non‐specific axial LBP +/− non‐dominant spine‐related leg pain and/or related neurological symptoms (e.g., paraesthesia tingling, pins and needles and/or numbness). Non‐dominant spine‐related leg symptoms are defined as the participant reporting their LBP being more bothersome than their leg symptoms.
d. Adequate English language proficiency and able to provide informed consent.
e. Booked for initial consultation at the Neurosurgery Spinal Assessment Clinic at either FSH or SCGH.
f. Willing to participate in both the pre‐ and post‐consultation interviews if deemed eligible for the post‐consultation interview following consultation with an ASP and subsequent discharge.

#### 2.5.2 Exclusion Criteria

a. Prior spinal surgery, including cervical, thoracic or lumbar spine surgery.
b. Diagnosed inflammatory condition affecting the spine, such as ankylosing spondylitis, rheumatoid arthritis or psoriatic arthritis.
c. Presence of symptoms suggestive of a dominant radicular syndrome, for example, lower limb pain described as sharp, burning, shocking, lancinating, electric, stabbing or shooting +/− reported tingling, pins and needles and numbness. Symptoms cover areas reminiscent of but not necessarily identical to dermatomes [27].
d. Presence of motor deficits likely to be related to radiculopathy, for example, reported foot drop [27].
e. Presence of symptoms suggestive of neurogenic claudication that dominates the clinical presentation, for example, participant reporting lower limb pain, paraesthesia, weakness and heaviness associated with walking and standing.
f. Involved in litigation or a work‐related injury with an active/open Workers' Compensation claim.
g. Inadequate English language proficiency or unable to provide informed consent for any reason.
h. Unwilling or unable to participate in both the pre‐ and post‐consultation interview if deemed eligible for post‐consultation interview following consultation with an ASP and subsequent discharge.

### 2.6 Eligibility Criteria Post‐Consultation Interview

#### 2.6.1 Inclusion Criteria

As per the pre‐consultation interview, the inclusion criteria plus:

a. Completed the pre‐consultation interview.
b. Attended initial consultation (+/− any required follow‐up consultation(s)) at the Neurosurgery Spinal Assessment clinic at either FSH or SCGH.
c. Discharged from an ASP clinic within 3 months of initial consultation without onward referral to a Consultant Surgeon's clinic.
d. Available for post‐consultation interview within 4 weeks of discharge.

#### 2.6.2 Exclusion Criteria

a. Onward referral for follow‐up in one of the Consultant Surgeon's clinics.
b. Deemed to have not met the pre‐consultation interview inclusion criteria or having met any of the pre‐consultation interview exclusion criteria following clinical examination by an ASP, for example, unexpected findings on clinical examination that could not be anticipated based on telephone eligibility screening.
c. Following clinical examination, a diagnosis of:
  ○ A specific spinal disorder, such as radiculopathy (with or without radicular pain) or spinal stenosis with neurogenic claudication, that dominates the clinical presentation.
  ○ A serious spinal disorder, such as cauda equina syndrome, infection, malignancy or fracture.

### 2.7 Post‐Consultation/Post‐Discharge Participant Selection

The ASPs involved in the patient's care will not know if their patient has consented to participate in the study. Therefore, ASPs will identify patients ‘potentially eligible for post‐consultation interview’ by: (i) having met Criteria a, b and c of the pre‐consultation inclusion criteria; (ii) not being excluded based on Criteria a–f of the pre‐consultation exclusion criteria and (iii) meeting the following post‐consultation eligibility criteria—discharged from their clinic within 3 months of initial consultation without onward referral to a Consultant Surgeon's clinic and without a diagnosis of a specific spinal disorder or serious spinal disorder as detailed above. As part of standard practice, the ASPs complete an ‘outcome slip’ following each patient consultation that goes to the neurosurgery clerk. For all discharged patients during the recruitment period, the ASPs will indicate on the outcome slip that the patient is potentially eligible for a post‐consultation interview. The ASPs will do this daily until informed by the CPI that the post‐consultation interview recruitment has finished.

The CPI will provide a list to the neurosurgery clerk at FSH and SCGH of participants from their hospital who have consented to the study and have completed the pre‐consultation interview and, therefore, are potentially eligible for the post‐consultation interview. This list will be used by the neurosurgery clerks to ‘match’ participants potentially eligible for the post‐consultation interview, as identified by the ASPs, to those who have undergone the pre‐consultation interview. The neurosurgery clerk at each hospital will contact, by email, the CPI and the researcher who conducted the pre‐consultation interview with a list of ‘matched’ participants ready and potentially eligible for a post‐consultation interview, at a minimum, on a weekly basis. The researcher who conducted the pre‐consultation interview will contact the participant by telephone to confirm the participant has been discharged/has no further follow‐up appointments to be booked and is available for a post‐consultation interview within the next 4 weeks, in which case a time to conduct the post‐consultation interview will be organised. Should a participant eligible for post‐consultation interview decline to partake in the post‐consultation interview, if possible, the reason for this will be ascertained and recorded, and consent to use data collected to date will be confirmed.

### 2.8 Data Collection

For pre‐consultation interviews, an initial five interviews will be conducted, followed by data analysis and then approximately 15–20 further interviews to find variants regarding perspectives to provide as broad an understanding of the issues as possible. This process will continue until no new (relevant) information can be found and additional sampling becomes redundant [26]. It is anticipated that 25 participants will be interviewed pre‐consultation, with approximately half from FSH and half from SCGH. However, the final numbers will be determined using the rationale above. Should an unequal number be recruited from the two sites, this will not impact the ability to address the aim of this study.

### 2.9 Semi‐Structured Interviews

The semi‐structured pre‐ and post‐consultation interview guide has been developed and partially informed by previous research [19, 28] and qualitative research experts (P.S. and C.B.) (Appendix). To develop the interview questions further, a consumer with LBP experience from Musculoskeletal Health Australia (https://muscha.org/) will be invited to comment on the questions.

The interview guide will initially be pilot tested by the CPI and researchers conducting the interviews and then by other members of the research team. Given the semi‐structured interview guide, the interviewer has the flexibility to probe participants to elaborate on specifics provided in their responses. All semi‐structured interviews will be conducted remotely via Zoom or telephone if required. Each interview is anticipated to last approximately 30 min. Participants will be informed that there is no obligation to answer (all) the questions and that they are free to leave the interview at any time. The ASPs who are responsible for the clinical care of the participants will not be conducting, nor will they be involved in, the semi‐structured interviews.

The pre‐consultation interview will focus on exploring participants' basic understanding of their LBP, their experiences while on the waitlist and their thoughts on ways to enhance care and make time spent waiting a more positive experience. Exploring participants' expectations pre‐consultation provides context for understanding how expectations shape the post‐consultation experience—offering an analysis of changes or alignment between expectations and outcomes. During the post‐consultation interview, the content of the ASP consultation(s) will be explored, that is, the overall experience and whether prior expectations were met. The post‐consultation interview will also explore experiences related to the outcomes of the consultation(s), such as advice provided (or not) and/or recommendations made (or not) and discharge pathways. Further, participants' reflections on their care journey and suggestions for improvement will be explored.

Capturing initial perspectives through the pre‐consultation interview helps minimise recall bias in the post‐consultation interview, where participants may forget or deviate from their earlier expectations. The pre‐consultation interview establishes baseline findings, enabling a more accurate comparison between participants' expectations and actual experiences, which strengthens the credibility and depth of the analysis. Some participants will be interviewed once (pre‐consultation only group), while others will be interviewed twice (pre‐ and post‐consultation group). The analysis will compare data from both groups, focusing on common themes related to experiences and expectations. Pre‐ and post‐consultation interview data will be linked as a paired dataset for participants interviewed twice, comparing each participant's expectations with their actual experiences. For this group with both pre‐ and post‐consultation interview data, we will examine any shifts or changes in understanding, expectations or thoughts on improving their care journey after the consultation, while the pre‐consultation‐only group will provide a snapshot at one point in time without comparison to later experiences. The core analysis will centre on identifying common themes across both groups, ensuring the study remains focused on participants' expectations and experiences.

Interviews will be video and audio recorded using online meeting (Zoom) software. Telephone interviews will be audio recorded. Only the audio component will be exported and transcribed verbatim by an experienced independent transcriptionist company (https://waywithwords.net). Audio recording will be coded so it can be linked to the demographic questionnaire data for the participant.

### 2.10 Participant Withdrawal Criteria

Participants can withdraw at any stage before analysis or withdraw any unprocessed data they have supplied without prejudice or penalty by contacting the CPI. Where possible, participants will be asked to sign the Withdrawal of Participation Form provided and return this to the CPI. Researchers will make a reasonable effort to ascertain the reason for withdrawal from the study, and where available, this will be recorded. However, participants are not obligated to provide a reason for withdrawal. Participants who withdraw will not be replaced after the data collection period. Participants will be reassured that withdrawal from the study will not influence their care or relationship with their care providers or other hospital staff involved in their current or future care, nor their relationship with the research investigators or CQUniversity (CQU).

### 2.11 Data Analysis

All transcripts will be reviewed for errors and corrected, and any potential identifying information will be removed before coding. Participants will be invited to member check their transcript by reviewing their transcript and making any necessary edits to ensure their responses accurately reflect their intended responses. They will be given 2 weeks to complete this task with a reminder after 1 week. No response will be assumed to indicate no issues with the transcript. One researcher (M.F.) will lead the qualitative analysis, and the other researchers (P.S. and C.B.) will independently review the analysis of a selection of the transcripts and reach consensus on the themes and categories from the data. Where discrepancies are discovered, research team members will meet and discuss these to achieve consensus. We will use an established checklist for study reporting, for example, Consolidated Criteria for Reporting Qualitative Research—COREQ [29].

Content analysis is considered the standard analysis strategy in qualitative descriptive studies [25]. We will conduct an *inductive content analysis* [30, 31] of the transcripts, which will involve coding and the generation of themes (categories). Themes with supporting quotations will be reported in a manuscript to summarise the data. Data organisation and coding will be supported by NVivo software (QSR International Pty Ltd, Melbourne, Australia).

### 2.12 Trustworthiness and Minimising Bias

To facilitate trustworthiness [32] and minimise potential bias, a minimum of two team members will independently review coding from the first set of transcripts and meet to come to a consensus and develop preliminary themes. A research team member will resolve any coding discrepancies/debates. Alternatively, in lieu of independent coding, we may have team member(s) conduct ‘audits’ of coding to see if it is indeed anchored in the data.

Several strategies will be used to minimise selection bias. First, all efforts will be made to post the study flyer to all consecutive patients with a booked initial consultation during the recruitment period. Second, all potential participants who contact the CPI expressing an interest in participating will be screened with the standardised eligibility screening checklist to determine whether they meet the eligibility criteria. The reason for exclusion at this stage will be recorded and reported as the proportion screened but not eligible. We will track and report the number of eligible participants (following telephone screening) who are sent the PICF and Qualtrics (Qualtrics, Provo, Utah, the United States) link to the consent form and demographics questionnaire (or posted hard copy versions) who, in the end, do not consent or never reply. Third, all patients who potentially meet the eligibility criteria for the post‐consultation interview will be identified to ensure no one who has consented and completed the pre‐consultation interview will miss out on the post‐consultation interview if eligible. At the completion of the study, the reason participants who completed the pre‐consultation interview were not eligible for the post‐consultation interview will be recorded and reported.

### 2.13 Informed Consent Process and Collection of Participant Characteristics

Following telephone screening, those eligible for the study who have an email address will be sent the PICF along with a Qualtrics link to an electronic consent form and demographics questionnaire. Once electronic consent is provided, the participant will complete the demographics questionnaire which includes age, sex, work status and postal code (to determine geographic remoteness), LBP duration, average pain intensity in the last week for both low back and (worst) leg using a numerical rating scale (0 = no pain and 10 = worst pain imaginable) [33], and the amount of limitation in daily activities experienced due to LBP (0 = no limitation at all and 10 = completely disabled) [34]. Potential participants who were screened for eligibility by telephone and agreed to receive the PICF and link to the electronic consent form and demographics questionnaire, who do not provide consent and complete the questionnaire within 1 week, will be followed up by telephone by a research assistant from CQU. This will help determine if they still wish to participate and answer any questions and remind them to access the link. A maximum of three attempts to contact potential participants by telephone will be made over a 1‐week period.

For potential participants without an email address, the PICF and a paper‐based version of the demographic questionnaire will be sent via post, along with a reply‐paid envelope to return the signed consent form and completed demographics questionnaire to the CPI. This information will be manually added to the Qualtrics software database by the RA at CQU, and consent will be recorded. Potential participants who require hard copy documents will be contacted by telephone 7 days after the documents have been posted to ensure the documents have been received, confirm they still wish to participate and answer any questions. This will help facilitate a timely return of the paper‐based consent forms so that the pre‐consultation interview can occur before the booked initial consultation appointment. It is anticipated that there will be very few, if any, hard copy consent forms and demographic questionnaires.

Referral characteristics of consented participants will be retrieved from the patient's hospital e‐referral system by the neurosurgery clerk at each hospital and provided to the CPI to be manually added to the Qualtrics software database. Referral characteristics to be collected include referral source, date referral received and date of initial consultation (to calculate wait time), initial triage category (expected clinical urgency and recommended time frames for new outpatient appointment), whether a triage category escalation was received and whether the participant had any follow‐up consultation(s) after initial consultation.

### 2.14 Data Management

Hard copy documents will be sent to a registered mailbox and stored in a locked cabinet at CQU in the CPI's office, with electronic data stored in CQU's dedicated research servers in accordance with CQU policy, with only CQU researchers involved in this study having access. This data will be retained in accordance with the Queensland Universities data retention and disposal schedule.

### 2.15 Risk Management

The research team has designed this study to minimise potential risks. There are no physical risks to participants or researchers as this study only involves interviews conducted remotely and the completion of a demographic questionnaire. There is a minor risk of inconvenience to participants due to the time commitment to complete the demographics questionnaire and to participate in one, and for some participants, two interviews. This is mitigated by the demographic questionnaire being short and easy to complete, the interviews lasting approximately 30 min each and being conducted via Zoom video conference or telephone, so there is no need for the participant to travel. There is also the possibility of inconvenience or time burden for participants who are invited to review their transcripts (although they do not have to if they don't want to). Participants will be informed verbally and in the PICF of the time commitment required, so they will be able to make an informed decision about voluntarily participating.

Secondly, there is a potential risk of emotional distress in participating in the interview(s). There is the possibility of emotional upset when participants discuss their LBP and its impact on their lives. Further, questions that generate a discussion of the consultation(s) may result in relief or alternatively, disappointment or frustration, that is, booked in an ASP‐led clinic, and the consultation did not extend beyond the ASP clinic, thus perceived to have no input from a surgeon. Further, questions regarding the discharge experience or expectations not being met at the consultation may upset study participants. This risk of emotional distress is mitigated by the interviewer first discussing the potential risks at the commencement of the interview and reassuring participants that they do not have to answer a question(s) if they do not want to or can stop the interview at any time. Participants will be informed over the telephone and in the PICF that they are welcome to have a family member or friend present during the interview if they wish (they will be asked not to share study information with others). Should a participant become distressed, the interviewer will stop the interview and attempt to alleviate the participant's distress by taking a break. The interview will only continue if the participant wishes to continue and the interviewer feels it is safe to proceed. If there is ongoing concern about the participants' mental health or well‐being, the participant will be encouraged to make an appointment with their General Practitioner (GP) and/or encouraged to contact Lifeline or Beyond Blue and/or appropriate support can be organised on behalf of the participant. If deemed necessary, the interviewer will directly contact the participant's GP, sharing their concerns. In the very unlikely event that the level of concern warrants a more immediate intervention, the interviewer will organise a welfare check at no cost to the participant. If the level of concern warrants emergency intervention, the interviewer will not hesitate to contact 000. It should be noted that the researchers conducting the interviews are healthcare professionals and have experience conducting interviews with clinical groups in health settings in other studies. All participants will be made to feel safe to disclose sensitive information and will be reassured that the information discussed will be de‐identified so individuals cannot be identified in any way by others outside of the person conducting the interview, the CPI and the RA at CQU.

Thirdly, there is a risk of breach of participant confidentiality/anonymity since participants will be interviewed and data collected will be re‐identifiable. This risk will be mitigated by all participant data being de‐identified by the CPI, with each participant given a unique Study ID on entry into the study. The re‐coding sheet to re‐identify data will be stored separately from the de‐identified data, with only the CPI and RA at CQU having access to identifiable data and the re‐coding sheet. The ASPs who are involved in the clinical care of the participants in this study will remain blinded to their participation while under their care.

Finally, it is possible that initial telephone screening for study eligibility could elicit clinically concerning symptoms that require review and/or action in an earlier time frame than the potential participant's booked clinical appointment with an ASP. Telephone screening for eligibility is being conducted by the CPI, who is a registered healthcare professional and therefore has the required knowledge and skills to interpret the responses and information provided by potential participants and take appropriate action. Depending on the level of concern and urgency, the CPI's action may include advising the patient to see their GP or attend their local Emergency Department. In all cases, the relevant hospital's PI who also works in the neurosurgery department will be notified and appropriate follow‐up ensured.

### 2.16 Dissemination of Findings

Results from this study will be submitted for peer‐reviewed publication and will be presented at relevant national and international conferences. Participants will be offered a layperson's summary of study findings, which will be emailed or posted to them.

### 2.17 Remuneration

Participants will receive a gift certificate via email or post after completing one or both interviews to thank them for their time.

## 3 Discussion

The intent of this protocol paper is to describe the study design and methodology rather than report results. Themes generated will shed light on the participants' experiences, expectations and care journey, collected both pre‐ and post‐consultation, within elective spinal surgery clinics in Western Australia. The pre‐consultation results from this study may provide insights regarding consultation expectations, such as initially high or desperate hopes, or limited understanding of their LBP, that may underpin low expectations about their role in their own care. Post‐consultation results may see a shift in perspectives, including expectations, which may evolve through the consultation process. This may range from relief that surgery is not required and a new mindset that focuses on self‐managing their LBP through various (lifestyle) modifications—such as health coaching and/or educational resources [35]. We also anticipate disappointment and/or frustration among some participants, particularly if surgery was seen as a ‘quick fix’ but was not offered or if their (LBP) experiences were not validated [18]. Previous studies have reported participants expressing feelings of being overlooked and therefore disheartened by their experience of consulting in a surgical service and being discharged without surgery [19]. This may lead to repeated consultations and sustained high healthcare use [20]. Overall, we foresee that our findings will resonate with prior research, where participants expected or hoped for urgent surgery and/or a definitive diagnosis following consultation in a surgical service [19]. The results of this study may give rise to consumer‐focused solutions to improve the care journey starting from the time spent waiting for the initial consultation, that is, providing education or exercise resources and/or other supports [36], as well as a better understanding of what is important to patients during their consultation(s). Finally, the results may also provide insight into non‐surgical management pathways or other supportive services that would best meet the needs of patients consulting in and discharged without surgery from elective spinal surgery clinics.

## 4 Reflexivity

We will keep a log of reflections and decision‐making throughout the research process (i.e., team member roles, interviewers' skills, personal biases and potential influence on participant responses, assumptions and experiences). While this would be completed when using the COREQ reporting items, we will document a reflective journal in the final manuscript to ensure transparency and rigour in our study.

## 5 Conclusions

This protocol describes the methodological process for a qualitative investigation into the experiences, expectations and care journey of LBP patients within elective spine surgery clinics in Western Australia. The data will provide participant accounts that may identify gaps in patient expectations and understanding of non‐surgical management for LBP, informing the development of tailored educational resources, communication strategies and alternative care pathways.

## Author Contributions

**Matthew Fernandez:** conceptualisation, investigation, funding acquisition, writing – original draft, writing – review and editing, methodology, project administration. **Deb Lees:** conceptualisation, writing – original draft, writing – review and editing. **Katie Luca:** conceptualisation, writing – original draft, methodology, writing – review and editing. **Dawn Dane:** conceptualisation, writing – original draft, writing – review and editing, methodology. **Peter Stilwell:** conceptualisation, writing – original draft, methodology, writing – review and editing. **Leanda McKenna:** conceptualisation, writing – original draft, writing – review and editing, methodology. **Brigitte Tampin:** conceptualisation, writing – original draft, writing – review and editing, methodology. **Caroline Bulsara:** conceptualisation, writing – original draft, writing – review and editing, methodology. **Tamar Pincus:** conceptualisation, writing – original draft, writing – review and editing, methodology. **Michelle Kendell:** conceptualisation, investigation, writing – original draft, writing – review and editing, methodology, project administration.

## Ethics Statement

Ethics approval is pending and in process for review by the WA Health Central Human Research Ethics Committee (Central HREC), and site approvals will be sought at FSH and SCGH before commencing the study. CQUniversity ethics will reciprocate the WA Health Central HREC approval. This study will comply with the National Health and Medical Research Council National Statement on Ethical Conduct in Human Research (‘National Health and Medical Research Council, Australian Research Council and Universities Australia (2023). National Statement on Ethical Conduct in Human Research. Canberra: National Health and Medical Research Council’). At the time of submitting this protocol for peer review, participant recruitment has not yet commenced.

## Consent

Patient consent is not required currently as the study has not yet commenced and is pending ethics approval.

## Conflicts of Interest

The authors declare no conflicts of interest.

## Supporting information

April APPENDIX interview questions Clean MF April MK.

## Data Availability

The authors have nothing to report.
