# Supplementary material for: ‘Before and After’. The Journey of Patients With Low Back Pain Consulting in Elective Spine Surgery Clinics. A Qualitative Study Protocol
Source: Health Expect. 2025 May 20;28(3):e70301. doi: 10.1111/hex.70301 (PMC12090200; doi:10.1111/hex.70301)
Supplement: Supplementary file 1 — April APPENDIX interview questions Clean MF April MK. [file HEX-28-e70301-s001.docx]

**APPENDIX: Interview questions**

The interview (guide) will be structured as follows.

**Pre-consultation questions:**

1. Thank you for completing the demographic questionnaire. You mentioned experiencing low back pain for [*time fame*]. Is that correct? To start: what do you understand might be causing your low back pain? (prompt might relate to “consider what your doctor or other professionals might have told you”).
2. I’d like to talk a little now about the time you have been on the waitlist for your initial consultation at the neurosurgery service (acknowledging that this has likely been a very long time …)

- What have been your experiences while waiting for you (initial) appointment?
- Do you feel your pain has changed during your time on the wait list? In what way?
- What have you been doing to manage the pain?
- What types of treatments or healthcare advice have you sought?

1. What do you think it will take or what is needed to help / improve your low back pain?

Do you think you personally can do anything to help / improve your low back pain?

- Do you need support from others or health care professionals to take these steps?

1. You have your consultation coming up. Do you have any expectations with respect to your upcoming consultation and if so, what would those be *(if yes or maybe*)?

4b. So, in terms of the possible outcomes following your consultation, have you thought about what you would do next to manage your low back pain problem if it cannot be helped with surgery?

- Do you expect to be offered different treatment options or told what steps you can take to manage your low back pain?

1. Do you have any thoughts on potential solutions for improving (or enhancing) your experiences while being on the waiting list for your first appointment?

- What could be done differently to make the experience a more positive one?
- What specific changes or information do you think would make the time spent waiting more useful or easier?

Is there anything else we have not covered today that you would like to comment on or add based on our conversation today?

**Post consultation / post-discharge questions:**

1. You recently attended your consultation(s) at the neurosurgery service. I realise it might be hard to remember all the details, but can you talk a little bit about your experiences during your consultation(s)? I have a few questions to help you recall your experiences…..

- Is there any thing that really stood out for you during your consultation?

2. Thinking back to the outcomes from your consultation(s), overall how do you feel? Let’s break it down a little (i) the initial assessment, (ii) follow up (if you had a follow up appointment), (iii) outcomes of the consultation(s), and (iv) discharge recommendations.

- Do you feel you got what you hoped for? If not, why not?
- What could have been done better?
- What could have been communicated better?

3. In our first interview, we talked about your expectations going in – based on your experience, do you feel your expectations were met?

- Why?
- Why not?

4. During your consultation, do you think your concerns were listened to and addressed?

- If yes: What was it about the consultation(s) that made you feel that you were listened to?
- If no: What concerns were not listened to/addressed/? How do you feel your concerns could have been better addressed?

5. Let’s talk a little about your experiences related to the outcomes of your consultation(s). For example, you may have been given some advice and/or recommendations (recommendations may include treatment options, a referral to another service / other healthcare professional [*prompts, for example, physiotherapy, chronic pain service*]) from the clinician you saw.

What advice and/or recommendations were you given during your consultation(s)?

- Do you feel you are able to follow through with this advice or the recommendations provided?
- If yes, what how will you go about achieving this?

If not, what in particular makes you feel that you are unable to? [Probe possible time / money / motivation / support / access to services to do so / other reasons?]

- Is there anything that could help you to be able to follow through?

5b. From what was explained, do you understand the reason(s) why you were discharged without surgery? Could you briefly explain your perspective to me?

5c. And thinking about your care journey through the neurosurgical service, how do you feel about having (specifically) been discharged without a surgical solution to your low back pain?

6. In looking back or reflecting on your entire care journey, from the time of your referral to your discharge from the service, what was it that made your care journey a positive or negative experience?

- Is there anything that could have been done differently or improved to make your experience a more positive one?

Do you have any other comments or thoughts on what we have talked about or anything we didn’t cover that you would like to add?
